# Drug Repurposing of Bromodomain Inhibitors as Potential Novel Therapeutic Leads for Lymphatic Filariasis Guided by Multispecies Transcriptomics

**DOI:** 10.1128/mSystems.00596-19

**Published:** 2019-12-03

**Authors:** Matthew Chung, Laura E. Teigen, Silvia Libro, Robin E. Bromley, Dustin Olley, Nikhil Kumar, Lisa Sadzewicz, Luke J. Tallon, Anup Mahurkar, Jeremy M. Foster, Michelle L. Michalski, Julie C. Dunning Hotopp

**Affiliations:** a Institute for Genome Sciences, University of Maryland School of Medicine, Baltimore, Maryland, USA; b Department of Microbiology and Immunology, University of Maryland School of Medicine, Baltimore, Maryland, USA; c Department of Biology, University of Wisconsin Oshkosh, Oshkosh, Wisconsin, USA; d New England Biolabs, Ipswich, Massachusetts, USA; e Greenebaum Cancer Center, University of Maryland, Baltimore, Maryland, USA; University of North Carolina at Charlotte

**Keywords:** lymphatic filariasis, filarial nematodes, nematodes, *Brugia malayi*, *Wolbachia*, *Aedes aegypti*, mosquito, transcriptomics, RNA-Seq, multispecies RNA-Seq, bromodomain, bromodomain inhibitor, 6S RNA, *Brugia*, drug repurposing, neglected tropical disease, symbiosis

## Abstract

To better understand the transcriptomic interplay of organisms associated with lymphatic filariasis, we conducted multispecies transcriptome sequencing (RNA-Seq) on the filarial nematode Brugia malayi, its *Wolbachia* endosymbiont *w*Bm, and its laboratory vector Aedes aegypti across the entire B. malayi life cycle. In *w*Bm, transcription of the noncoding 6S RNA suggests that it may be a regulator of bacterial cell growth, as its transcript levels correlate with bacterial replication rates. For A. aegypti, the transcriptional response reflects the stress that B. malayi infection exerts on the mosquito with indicators of increased energy demand. In B. malayi, expression modules associated with adult female samples consistently contained an overrepresentation of genes involved in chromatin remodeling, such as the bromodomain-containing proteins. All bromodomain-containing proteins encoded by B. malayi were observed to be upregulated in the adult female, embryo, and microfilaria life stages, including 2 members of the bromodomain and extraterminal (BET) protein family. The BET inhibitor JQ1(+), originally developed as a cancer therapeutic, caused lethality of adult worms *in vitro*, suggesting that it may be a potential therapeutic that can be repurposed for treating lymphatic filariasis.

**IMPORTANCE** The current treatment regimen for lymphatic filariasis is mostly microfilaricidal. In an effort to identify new drug candidates for lymphatic filariasis, we conducted a three-way transcriptomics/systems biology study of one of the causative agents of lymphatic filariasis, Brugia malayi, its *Wolbachia* endosymbiont *w*Bm, and its vector host Aedes aegypti at 16 distinct B. malayi life stages. B. malayi upregulates the expression of bromodomain-containing proteins in the adult female, embryo, and microfilaria stages. *In vitro*, we find that the existing cancer therapeutic JQ1(+), which is a bromodomain and extraterminal protein inhibitor, has adulticidal activity in B. malayi.

## Abstract (precis)

The current treatment regimen for lymphatic filariasis is mostly microfilaricidal. In an effort to identify new drug candidates for lymphatic filariasis, we conducted a three-way transcriptomics/systems biology study of one of the causative agents of lymphatic filariasis, Brugia malayi, its *Wolbachia* endosymbiont *w*Bm, and its vector host Aedes aegypti at 16 distinct B. malayi life stages. B. malayi upregulates the expression of bromodomain-containing proteins in the adult female, embryo, and microfilaria stages. *In vitro*, we find that the existing cancer therapeutic JQ1(+), which is a bromodomain and extraterminal protein inhibitor, has adulticidal activity in B. malayi.

## INTRODUCTION

Lymphatic filariasis is spread by mosquitoes infected with one of three filarial nematodes: Wuchereria bancrofti, Brugia malayi, or Brugia timori. As of 2016, an estimated 856 million individuals are living in areas with ongoing transmission of the disease (1). Infected individuals are often asymptomatic with subclinical parasite-associated immunosuppression (2), while approximately one-third of affected individuals manifest symptoms such as chronic lymphedema and/or hydrocele, both caused by the damage inflicted by the nematodes on the human lymphatics. Although the disease is rarely fatal, >25 million affected individuals are afflicted with hydrocele and >15 million individuals are afflicted with lymphedema (1).

Mass drug administration (MDA) programs aimed at eradicating the causative agents of lymphatic filariasis employ a combination of diethylcarbamazine, doxycycline, ivermectin (IVM), and/or albendazole (3–6). Although each of the drugs has a different mechanism of action (7), diethylcarbamazine, ivermectin, and albendazole are primarily microfilaricidal (6, 8–10), with only doxycycline having significant adulticidal activity (11).

Although current MDA programs have been successful in lowering transmission of lymphatic filariasis (1), the limited adulticidal efficacy of current drugs indicates a need for novel therapeutics. The obligate relationship between B. malayi and its bacterial *Wolbachia* endosymbiont *w*Bm has provided one of the more recent novel targets in filarial nematode elimination efforts (13–15), since inhibiting *w*Bm halts development and reproduction in B. malayi and, more importantly, kills adult worms. To this end, numerous studies have used genome functional annotations (16–18) and differential expression analyses (19, 20) to identify potential drug targets in both B. malayi and *w*Bm.

Intraperitoneal infection of Mongolian gerbils by Brugia malayi is a frequently used system for studying lymphatic filariasis, since all mammalian life stages of the nematode can be isolated in the laboratory from a small rodent (21). Several transcriptomics studies have been conducted on the filarial worm, its bacterial endosymbiont, and/or its vector using this system (19, 20, 22), but no comprehensive study has been able to simultaneously analyze the transcriptome of all three organisms across the entirety of the B. malayi life cycle. One of the main reasons is the inability to recover a sufficient number of reads to represent the transcriptome of *w*Bm, which has a very low relative abundance in several key life stages. To address this, we used rRNA and poly(A) depletions (23) and Agilent SureSelect (AgSS) transcriptome capture (24) to enrich for *Wolbachia* reads for selected samples.

Here, we report the transcriptomes of B. malayi, *w*Bm, and its laboratory vector, Aedes aegypti, across the entire B. malayi life cycle, to better understand the biological interplay between the organisms. We expect this to be a community resource and as such have provided access to the data at the gene level for B. malayi, http://gcid.igs.umaryland.edu/pub/bm-RNA-life; *w*Bm, http://gcid.igs.umaryland.edu/pub/wb-RNA-life; and A. aegypti, http://gcid.igs.umaryland.edu/pub/aa-RNA-life. In B. malayi, chromatin remodeling seems to be a key activity in adult females, embryos, and microfilariae such that bromodomain inhibitors were examined as a potential therapeutic for lymphatic filariasis.

## RESULTS

### B. malayi transcriptome.

**FIG 1:**
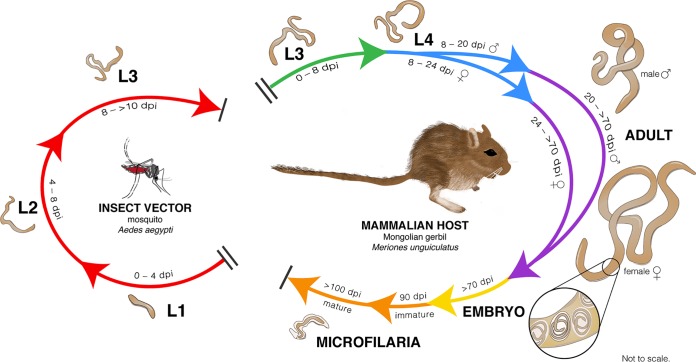
Brugia malayi life cycle and sample selection. Initial larval development (microfilaria/L1, L2, and L3) takes place in the thorax of the mosquito vector after ingestion of microfilaremic blood from the mammalian host. Subsequent development of L3 to L4 and finally to the mature adult occurs upon introduction of L3 to the mammalian host, in this case in the peritoneal cavity of the Mongolian gerbil. Life cycle stages sampled from mosquitoes included microfilariae that had penetrated the mosquito midgut and entered thorax muscle (18 hpi), molt to L2 larvae (4 dpi) and to L3 larvae (8 dpi) in mosquito thorax, and vector-derived infective L3 from the mosquito head. Life cycle stages sampled from gerbils included early L3 infection (1 to 4 dpi), molt to L4 larvae (8 dpi), molt of males (20 dpi) and females (24 dpi) to immature adults, sexually mature adults (>70 dpi), eggs and embryos from adult females, immature microfilariae not yet infective to mosquitoes, and mature infective microfilariae.

**FIG 2:**
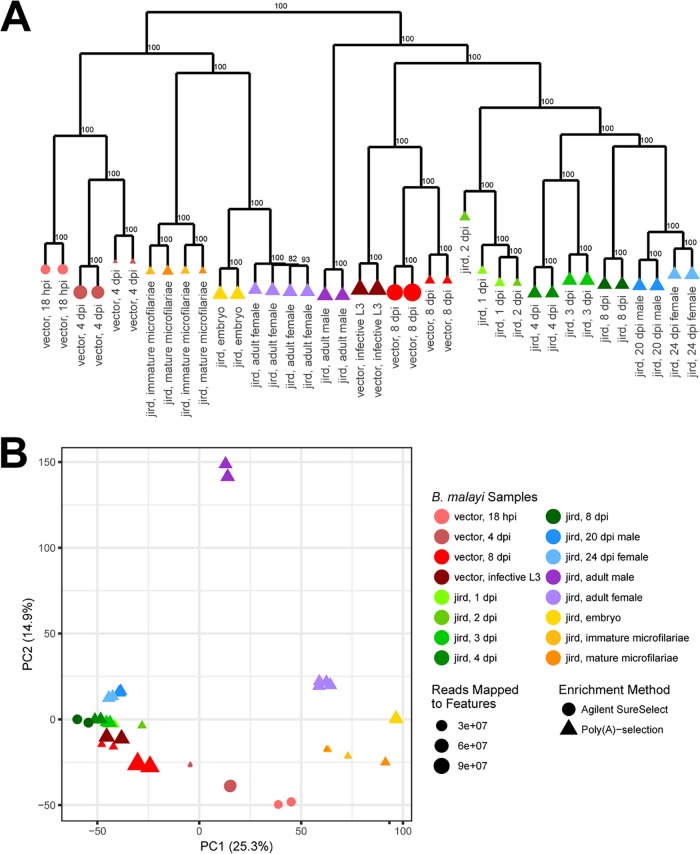
Hierarchical clustering and PCA of differentially expressed B. malayi genes. (A) For each sample, the z-scores of the log_2_(TPM) values of the 10,297 differentially expressed B. malayi genes were used to hierarchically cluster the B. malayi samples using 100 bootstraps, with support values indicated adjacent to the nodes. (B) A PCA was done using the z-score of the log_2_(TPM) values of the 10,297 differentially expressed B. malayi genes. The variation represented by each of the first two principal components is indicated in parentheses next to the axis titles. In both the hierarchical clustering and PCA, four clusters are observed. Samples represented by triangles were prepared using a poly(A) selection, while samples represented by circles were prepared using a B. malayi*-*specific AgSS capture. Points are sized relative to the number of reads mapping to genes for each sample. The B. malayi samples cluster based on sample rather than library enrichment, with all samples enriched using the AgSS clustering with their respective poly(A)-selected counterparts rather than each other.

**FIG 3:**
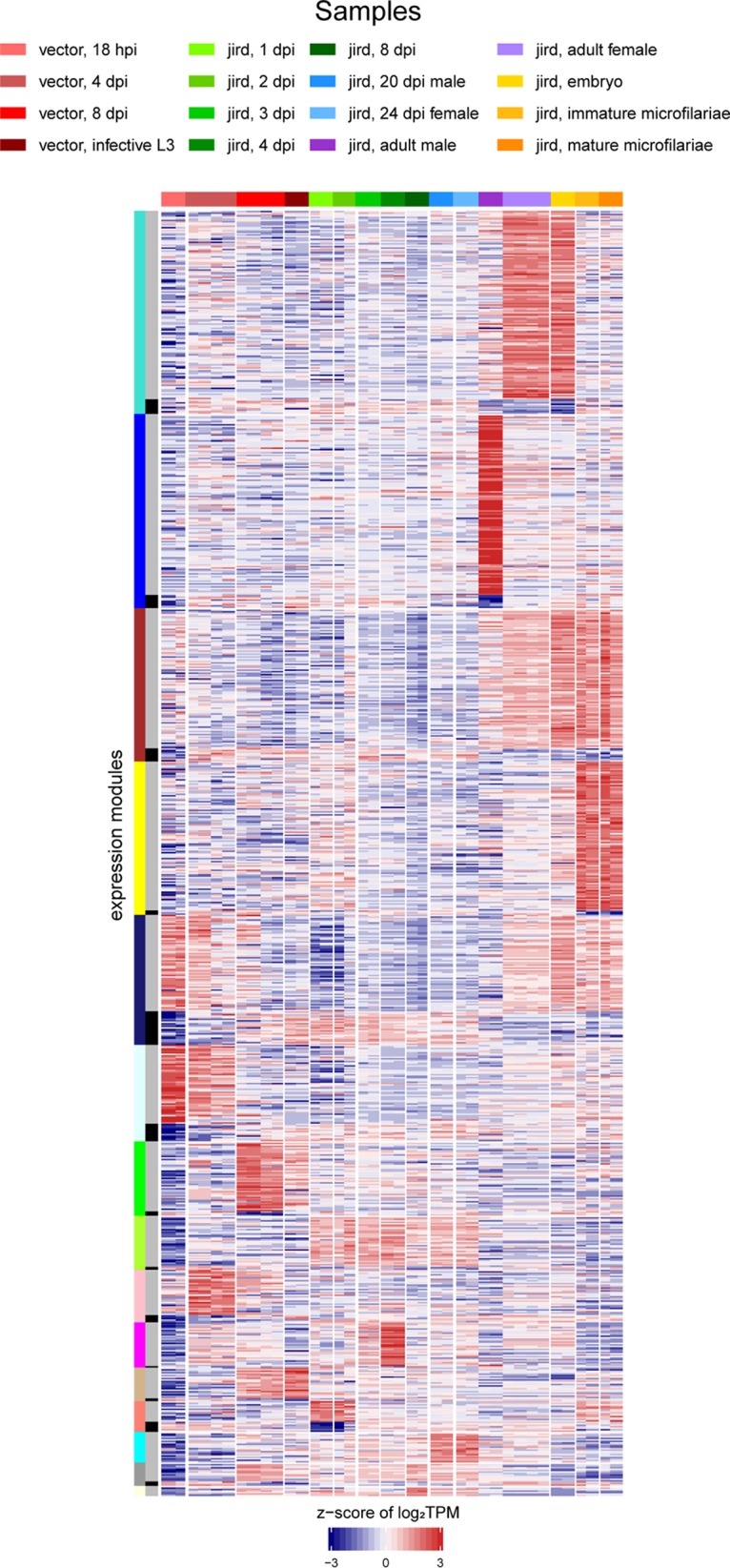
B. malayi WGCNA expression modules. The heatmap shows the z-score of the log_2_(TPM) values of the 10,297 differentially expressed B. malayi genes across the B. malayi life cycle. The horizontal color bar and the key above the heatmap indicate the samples represented in each of the heatmap columns. The leftmost vertical color bar on the left of the heatmap represents the 15 different WGCNA expression modules generated using the 10,297 differentially expressed B. malayi genes. Within each individual expression module, there are two expression patterns that are the opposite of each other. The inner vertical colored bar indicates whether the gene clusters with the main (gray) or inverse (black) expression pattern of the module.

A total of 16 distinct B. malayi life cycle stages were sampled and sequenced to assess the B. malayi transcriptome (Fig. 1; Table 1). Rarefaction curves generated from each of the samples indicate that the samples are sequenced to sufficient depth to accurately represent the B. malayi transcriptome, with the exception of the poly(A)-selected vector 18-h-postinfection (hpi) samples (see Fig. S1 in the supplemental material), which were excluded from subsequent analyses. Of the 11,085 predicted B. malayi protein-encoding genes, 10,207 (92.1%) had statistically significant differential gene expression across the life cycle. A hierarchical clustering and principal-component analysis (PCA) of these differentially expressed genes separates the samples into four distinct clusters of (A) the adult male samples; (B) the adult female, embryo, immature microfilaria, and mature microfilaria samples; (C) the L1 and L2 samples; and (D) the L3 samples from both the vector and the gerbil, all L4 samples, and the immature adult samples (Fig. 2). The 10,207 differentially expressed genes were clustered into 15 expression modules using WGCNA (25) (Fig. 3 and Fig. S2), with each cluster being split into a primary expression profile and a secondary, inverse expression profile. A functional term enrichment analysis was done on each of the WGCNA expression modules.

**TABLE 1:** RNA-Seq life cycle samples and library preparations

Nematode host and sample	B. malayi	*w*Bm	A. aegypti
Gerbil			
1 dpi	Poly(A) selection	rRNA, poly(A) depletion	NA^*a*^
2 dpi	Poly(A) selection	rRNA, poly(A) depletion	NA
3 dpi	Poly(A) selection	rRNA, poly(A) depletion	NA
4 dpi	Poly(A) selection	rRNA, poly(A) depletion	NA
8 dpi	Poly(A) selection	rRNA, poly(A) depletion	NA
20-dpi male	Poly(A) selection	rRNA, poly(A) depletion	NA
24-dpi female	Poly(A) selection	rRNA, poly(A) depletion and *w*Bm AgSS	NA
Adult male	Poly(A) selection	rRNA, poly(A) depletion	NA
Adult female	Poly(A) selection	rRNA, poly(A) depletion and *w*Bm AgSS	NA
Embryo	Poly(A) selection	rRNA, poly(A) depletion	NA
Immature microfilariae	Poly(A) selection	rRNA, poly(A) depletion	NA
Mature microfilariae	Poly(A) selection	rRNA, poly(A) depletion	NA
Mosquito			
18 hpi	B. malayi AgSS	*w*Bm AgSS	Poly(A) selection
4 dpi	Poly(A) selection and B. malayi AgSS	*w*Bm AgSS	Poly(A) selection
8 dpi	Poly(A) selection and B. malayi AgSS	*w*Bm AgSS	Poly(A) selection
Infective L3	Poly(A) selection	NA	Poly(A) selection

a NA, not applicable.

10.1128/mSystems.00596-19.1 FIG S1 Rarefaction curves for the B. malayi samples. (a) A rarefaction curve was generated by taking a subset of counts for each of the B. malayi samples and determining the number of protein-encoding genes able to be detected at each subset step. Each rarefaction curve is labeled by a color corresponding to a life stage sample and a shape corresponding to the library enrichment method. The circular/triangular points at the end of each curve represents the total number of protein-encoding genes detected with the full counts of the sample. Download FIG S1, TIF file, 10.8 MB. Copyright © 2019 Chung et al. 2019 Chung et al. This content is distributed under the terms of the Creative Commons Attribution 4.0 International license.

10.1128/mSystems.00596-19.2 FIG S2 Heatmap of the log_2_(TPM) of the differentially expressed B. malayi genes. The heatmap shows the log_2_(TPM) values of the 10,398 differentially expressed B. malayi genes across the B. malayi life cycle. The horizontal color bar and the key above the heatmap indicate the samples represented in each of the heatmap columns. The leftmost vertical color bar on the left of the heatmap represents the 15 different WGCNA expression modules generated using the 10,398 differentially expressed B. malayi genes. Within each individual expression module, there are two expression patterns that are the opposite of each other. The inner vertical colored bar indicates whether the genes cluster with the main (gray) or inverse (black) expression pattern of the module. Download FIG S2, TIF file, 2 MB. Copyright © 2019 Chung et al. 2019 Chung et al. This content is distributed under the terms of the Creative Commons Attribution 4.0 International license.

**(i) Adult males.** The second largest expression module identified in B. malayi consists of 1,442 genes upregulated and 99 genes downregulated in adult male B. malayi worms (Fig. 3). PapD-like major sperm proteins along with kinases and phosphatases were significantly overrepresented in genes upregulated in adult male B. malayi, as previously described (20). Between 30 and 40% of proteins with kinase and phosphatase domains were upregulated in males, likely indicative of male-specific signaling cascades in B. malayi. Adult male nematodes also upregulate an overabundance of proteins containing BTB/Kelch-associated domains. The Kelch repeats of these proteins have been found to form a β-propeller that interacts with actin and intermediate filaments for cytoskeletal rearrangement, while the BTB domains have been found to frequently interact with the Cul3 family of ubiquitin ligases (26).

**(ii) Adult females, embryos, and microfilariae.** There are five B. malayi expression modules containing genes differentially expressed in combinations of the adult female, embryo, and/or microfilaria life stages. The largest coexpression module identified in our data set includes 1,493 upregulated and 119 downregulated genes in the gravid adult female and embryo B. malayi life stages (Fig. 3). Because the bodies of gravid adult females are filled with embryos, the transcriptional profile of adult female worms is likely dominated by the transcriptional profile of eggs and embryos. The upregulated genes were found to be significantly overrepresented in proteins associated with nuclear localization and DNA replication, including all six proteins of the minichromosome maintenance complex responsible for initiating DNA replication.

The third largest cluster contains 1,118 upregulated and 99 downregulated genes in adult females, embryos, and microfilariae (Fig. 3). The upregulated genes contained are overrepresented in genes with protein binding, zinc fingers, and armadillo-type domains and also transcriptional regulators. The overrepresentation in zinc fingers include PHD-type zinc fingers, which have been found to have roles in epigenetic modifications and chromatin remodeling (27). We also observed an overrepresentation in genes encoding proteins containing bromodomains, a protein fold known for binding to acetylated lysines on histones to facilitate chromatin remodeling.

The fourth largest cluster contains genes differentially regulated in only microfilariae, including 1,172 upregulated and 42 downregulated genes with no overrepresentation of functional terms (Fig. 3). The hierarchical cluster analysis of the B. malayi samples shows the immature and mature microfilaria samples to be indistinguishable from one another (Fig. 2A), indicating that there is no significant difference in their transcriptomes. In the filarial nematode Onchocerca volvulus, the overrepresentation of G-protein-coupled receptors (GPCRs) in the microfilarial life stages is proposed to meet unique chemosensory requirements needed for the migration of the microfilariae to the bloodstream of their definitive host for uptake by a vector host (28). While not significantly overrepresented, 19 of the 80 (28.3%) B. malayi-encoded GPCRs are upregulated and in this module. Additionally, we see an overrepresentation of genes encoding proteins containing homeobox domains, a protein known to be involved in binding to DNA to facilitate eukaryotic development.

The fifth largest cluster included genes differentially regulated from the adult female through the L2 life stage with 768 upregulated and 265 downregulated genes (Fig. 3). Of the identified overrepresented functional terms for upregulated genes, chromatin remodeling is a recurring theme, containing 21 of the 74 B. malayi-encoded superfamily 1 and 2 [SF1/2] helicases [29]. The upregulated genes were also enriched for RNA recognition motifs, nucleic acid binding, ATP binding, DEAD/DEAH helicase domains, RNA processing, and protein binding while the downregulated genes were enriched for electron carrier activity.

**(iii) Vector stages L1 and L2.** Of the four B. malayi vector stages, the L1 and L2 nematode samples taken at 18 hpi and 4 days postinfection (dpi) share similar expression profiles (Fig. 2) with a cluster of 621 genes that were upregulated and 146 genes that were downregulated (Fig. 3). In the 621 upregulated genes, there is an overrepresentation of genes involved in protein production, cleavage, and degradation, suggesting a large turnover of proteins associated with the mammal to vector host transition. These include ribosomal components and proteins with translational roles, including translation initiation factors, and proteins with roles in tRNA aminoacylation and RNA binding. This cluster also includes the α/β proteasomal subunits, which function in peptide cleavage and protein degradation. This suggests that as nematodes transition from the mammal to the insect host, the dominant process becomes facilitating rapid protein turnover.

**(iv) Vector-derived L3.** There are two modules that represent genes upregulated later in B. malayi development in the vector host. The larger module contains 558 genes that are more highly upregulated in the 8-dpi samples (Fig. 3), while the smaller module contains 253 genes that are more highly upregulated in the infective L3 samples (Fig. 3). The larger module is overrepresented by genes encoding proteins with roles in ion transport, malate metabolism, and proteolysis, while the smaller module is overrepresented with proteins in the neuropeptide signaling pathway. Combined, these two modules contain 15 of the 80 differentially expressed GPCRs, which could serve as chemokine receptors that direct fully developed L3 to the mosquito head in preparation for a blood meal. Additionally, the smaller module contains 7 of the 10 B. malayi abundant larval transcripts (ALTs), encoding a class of larva-specific proteins, whose expression levels peak at the infective L3 stage and drop upon entry into the definitive host. Although ALTs have unknown and likely varying functions, they are promising vaccine targets for filariasis because they are larva specific and highly expressed and have no mammalian homologs (30).

**(v) Rodent-derived L3 and L4 and early adults.** There are 403 B. malayi genes upregulated 1, 2, 3, 4, 8, 20, and 24 days after infection of the gerbil, which represent a wide diversity of life stages and molts, including the L3, L4, and sexually immature adult life stages (Fig. 3). The only overrepresented functional terms identified describe membrane proteins and proteins with thioredoxin domains. A module was also recovered containing 168 upregulated and 84 downregulated genes at only 1 and 2 days after infection of the gerbil host (Fig. 3), indicative of the transcriptional alterations during the transition to the vertebrate host, with the upregulated genes being overrepresented with proteins involved with translation.

At 4 days after infection of the gerbil, the cuticle surrounding the head thickens in anticipation of the L3-to-L4 molt at 8 dpi (21). There is a module containing 411 genes upregulated at 4 dpi (Fig. 3) that is overrepresented in collagen triple helix repeats, nematode cuticle collagens, calycins, PAN/Apple domains, and zona pellucida domains. The third B. malayi molt occurs 8 days after infection of the gerbil with the secretion of a new collagenous cuticle (21). The smallest WGCNA module contains 74 genes upregulated at 8 dpi (Fig. 3) and has an overrepresentation of structural constituents of the nematode cuticle and collagen triple helix repeats.

Once L4 larvae have matured to early adults, at 20 dpi for the males and 24 dpi for the females, 238 genes are upregulated (Fig. 3) with an overrepresentation in transthyretin-like proteins that localize to extracellular space. No expression modules differentiating the 20-dpi male and 24-dpi female samples were recovered, despite the development of gonads.

### *w*Bm transcriptome.

**FIG 4:**
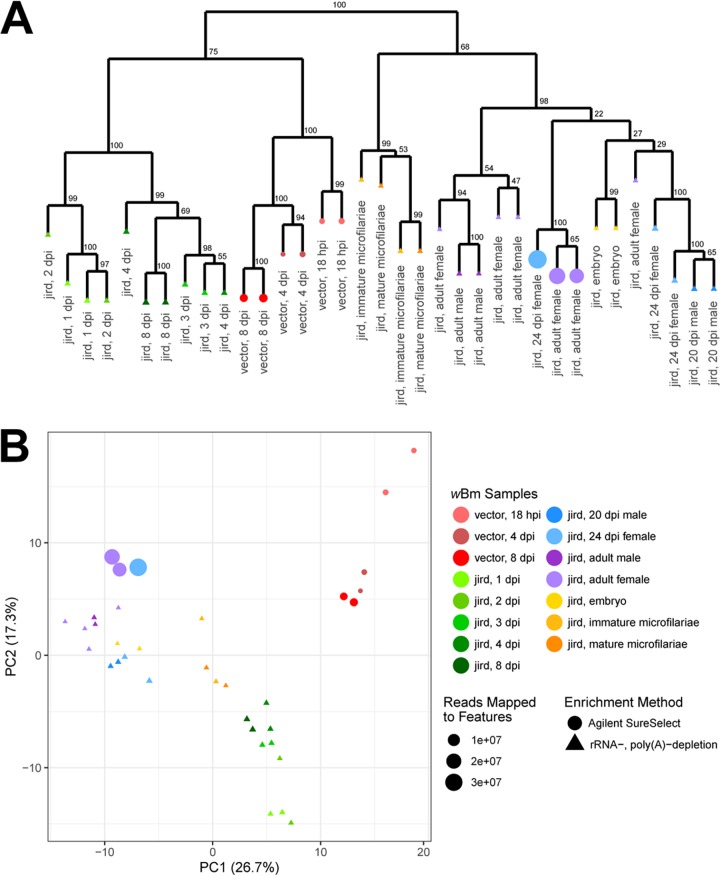
Hierarchical clustering and PCA of the differentially expressed *w*Bm genes. (A) For each sample, the z-scores of the log_2_(TPM) values of the 318 differentially expressed *w*Bm genes were used to hierarchically cluster the *w*Bm samples using 100 bootstraps with support values indicated at each node. Samples represented by triangles were prepared using an rRNA and poly(A) depletion, while samples represented by circles were prepared using a *w*Bm-specific AgSS capture performed on total RNA. (B) A PCA was performed using the z-score of the log_2_(TPM) values of the 318 differentially expressed *w*Bm genes. The first and second principal components are plotted on the *x* and *y* axes, respectively. The variation represented by each of the principal components is indicated in parentheses next to the axis titles. Points are sized relative to the number of reads mapping to genes for each sample.

The *w*Bm transcriptome was assessed for all the same time points as B. malayi with the exception of the infective L3 life stage. For each of the *w*Bm samples, sequencing libraries were prepared from rRNA- and poly(A)-depleted RNA with the exception of the samples originating from the vector host, two adult female samples, and a 24-dpi female sample, which were prepared using Agilent SureSelect transcriptome captures. Rarefaction curves for our *w*Bm data set show that all *w*Bm samples are at or near saturation (Fig. S3). Of the 839 annotated *w*Bm protein-encoding genes, 318 (37.9%) were identified to be differentially expressed in at least one B. malayi life stage. Despite some prior studies observing strong shifts in *Wolbachia* gene regulation between life stages, our hierarchical analysis and PCA using differentially expressed genes reveal poorly defined clusters (Fig. 4 and Fig. S4) with weak effects.

10.1128/mSystems.00596-19.3 FIG S3 Rarefaction curves for the *w*Bm samples. (a) A rarefaction curve was generated by taking a subset of counts for each of the *w*Bm samples and determining the number of protein-encoding genes able to be detected at each subset step. Each rarefaction curve is labeled by a color corresponding to a life stage sample and a shape corresponding to the library enrichment method. The circular/triangular points at the end of each curve represents the total number of protein-encoding genes detected with the full counts of the sample. Download FIG S3, TIF file, 1.3 MB. Copyright © 2019 Chung et al. 2019 Chung et al. This content is distributed under the terms of the Creative Commons Attribution 4.0 International license.

10.1128/mSystems.00596-19.4 FIG S4 Heatmap of the log_2_(TPM) of the differentially expressed *w*Bm genes. The heatmap shows the log_2_(TPM) values of the 336 differentially expressed *w*Bm genes across the B. malayi life cycle. The horizontal color bar and the key above the heatmap indicate the samples represented in each of the heatmap columns. The leftmost vertical color bar on the left of the heatmap represents the nine different WGCNA expression modules generated using the 336 differentially expressed B. malayi genes. Within each individual expression module, there are two expression patterns that are the opposite of each other. The inner vertical colored bar indicates whether the genes cluster with the main (gray) or inverse (black) expression pattern of the module. Download FIG S4, TIF file, 1.1 MB. Copyright © 2019 Chung et al. 2019 Chung et al. This content is distributed under the terms of the Creative Commons Attribution 4.0 International license.

**FIG 5:**
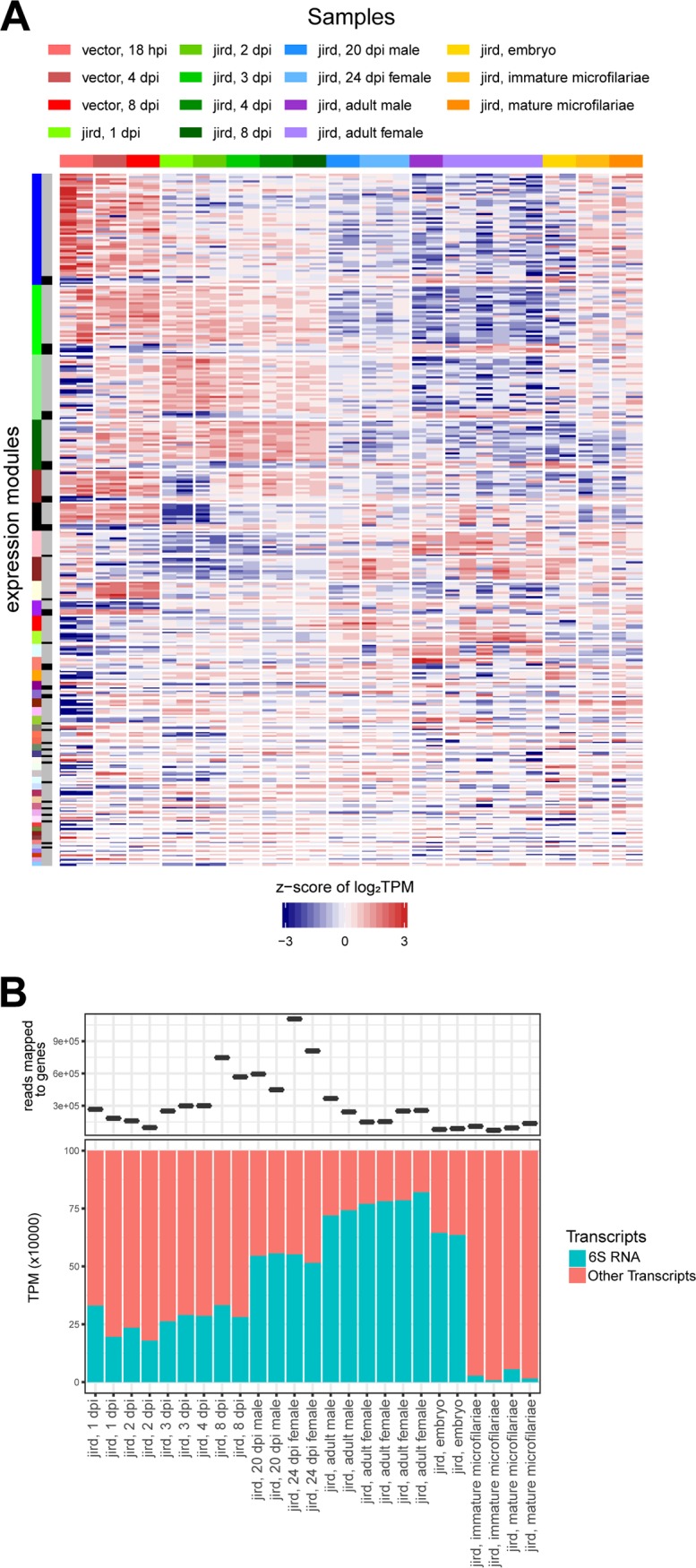
*w*Bm WGCNA expression modules and 6S RNA expression. (A) The heatmap shows the z-score of the log_2_(TPM) values of the 318 differentially expressed *w*Bm genes across the B. malayi life cycle. The horizontal color bar and the key above the heatmap indicate the samples represented in each of the heatmap columns. The leftmost vertical color bar on the left of the heatmap represents the six different WGCNA expression modules generated using the differentially expressed *w*Bm genes. The inner vertical colored bar indicates whether the genes cluster with the main (gray) or inverse (black) expression pattern of the module. (B) The upper plot indicates the number of reads mapped to genes in each of the samples. The bottom plot indicates the TPM value of the 6S RNA compared with that of all other transcripts for all poly(A)-selected samples. The Agilent SureSelect samples are excluded from the plot because there were no probes designed to capture the non-protein-encoding RNAs, such as the 6S RNA. Additionally, one replicate of 4 dpi underwent column purification, leading to the loss of small RNAs, and is not shown.

Using WGCNA, the 318 differentially expressed genes were clustered into 45 expression modules, with only 8 modules having ≥10 genes. The largest *w*Bm expression module contains 47 upregulated and 4 downregulated genes differentially expressed in vector samples (Fig. 5A), with no significantly overrepresented functional terms. Additionally, another cluster containing 13 genes upregulated in specifically the vector life stages (Fig. 5A) is enriched for the transcription factors containing GTP-binding domains.

The second largest cluster contains 27 upregulated and 5 downregulated genes in the vector and early gerbil samples (Fig. 5A), with the upregulated genes being enriched for translation and structural constituents of the ribosome. A cluster containing 12 genes upregulated in the vector stages and the jird 3-, 4-, and 8-dpi samples (Fig. 5A) is enriched for genes with roles in metabolic processes. While we see no further enriched functional terms in our larger *w*Bm expression modules, we do observe an enrichment of translational proteins in 8 genes upregulated specifically in the 4-dpi and 8-dpi vector stages (Fig. 5A). Similarly, we observe an enrichment of proteins with roles in unfolded protein binding and protein folding in 5 genes upregulated in the adult male and female samples (Fig. 5A). This is different than the prior observation of an overrepresentation of chaperones in only adult female B. malayi (19). Despite the physiological differences between adult male and female worms, there is little discernible difference in the *w*Bm transcriptomes of males and females in the hierarchical clustering or PCA patterns in our analysis (Fig. 4).

### 6S RNA.

This analysis of global gene regulation was conducted on only coding sequences (CDSs), precluding observation of differentially expressed noncoding RNAs, like the 6S RNA. However, across the life stages there is a strong difference in expression of the 6S RNA, a noncoding RNA whose expression correlates with bacterial replication rates (31, 32). In *w*Bm, the expression of the 6S RNA appears to correlate with *Wolbachia* titer (33, 34), with the expression of the 6S RNA increasing as B. malayi matures from the L3 to the adult and embryo life stages and dropping precipitously upon the embryo maturing to microfilaria (Fig. 5B).

### A. aegypti transcriptome.

**FIG 6:**
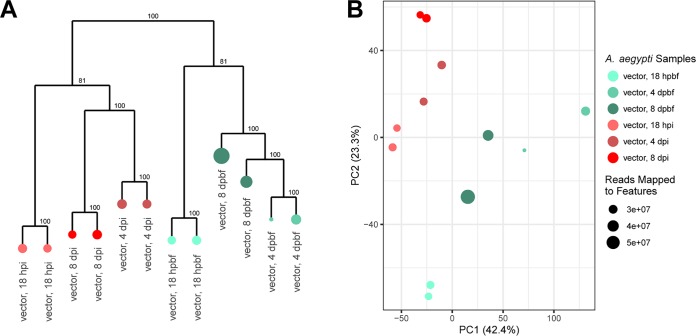
Hierarchical clustering and PCA of the differentially expressed A. aegypti genes. (A) A hierarchical clustering analysis of the z-score of the log_2_(TPM) values of the 6,615 differentially expressed A. aegypti genes was used to cluster the A. aegypti samples. Post-blood-feeding controls (hpbf and dpbf) were obtained and used as a control for the postinfection samples at the same time interval (hpi and dpi). Support values were calculated using 100 bootstraps and are indicated adjacent to the nodes. (B) A PCA was performed using the z-score of the log_2_(TPM) values of the 6,615 differentially expressed A. aegypti genes with the variation indicated in parentheses next to the axis titles. Points are sized relative to the number of reads mapping to genes for each sample.

**FIG 7:**
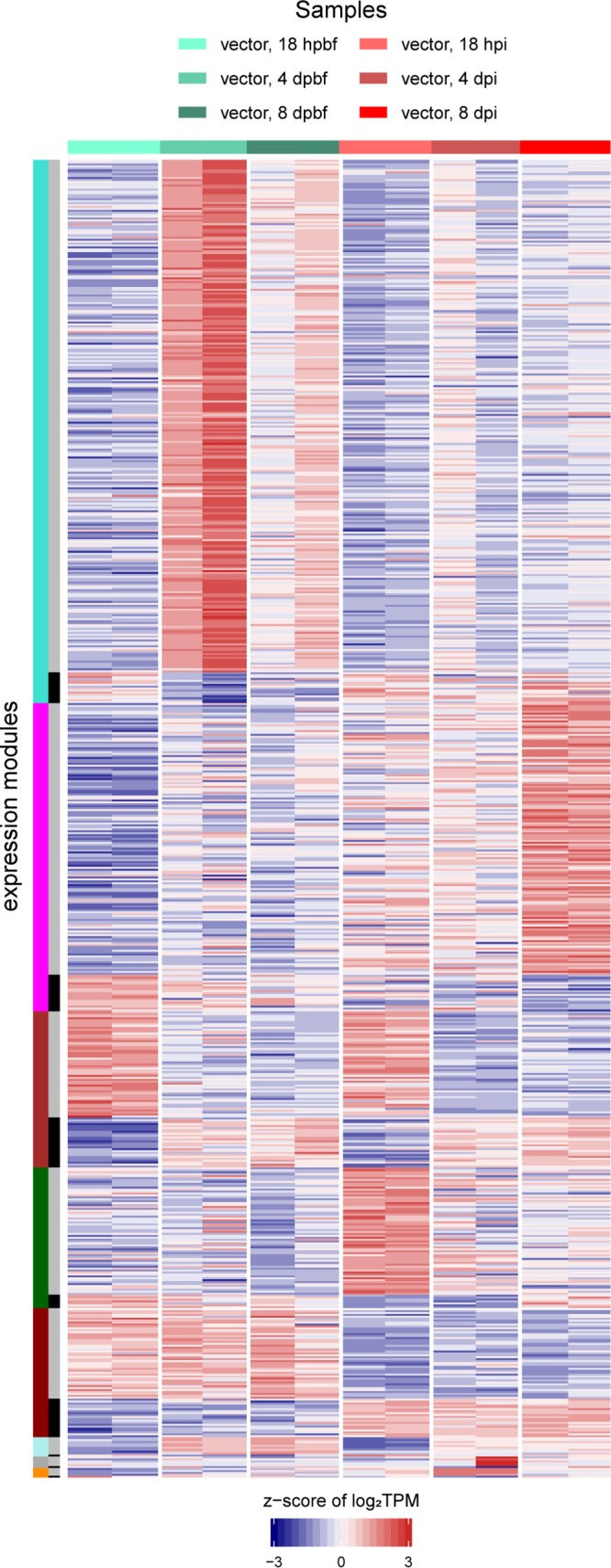
A. aegypti WGCNA expression modules. The heatmap shows the z-score of the log_2_(TPM) values of the 6,615 differentially expressed A. aegypti genes. The horizontal color bar and the key above the heatmap indicate the samples represented in each of the heatmap columns. The leftmost vertical color bar on the left of the heatmap represents the nine different WGCNA expression modules. The inner vertical colored bar indicates whether the genes cluster with the main (gray) or inverse (black) expression pattern of the module.

The transcriptomes of A. aegypti 18 h, 4 days, and 8 days after B. malayi infection were compared to the transcriptomes from time-matched controls that were fed uninfected dog blood. Of the 17,313 protein-encoding genes annotated in A. aegypti, 6,615 (38.2%) were found to be differentially expressed under at least one of the six conditions. Hierarchical clustering and PCA show distinct clustering of infected and control samples (Fig. 6). A rarefaction curve indicates that we sequenced all A. aegypti samples sufficiently to accurately represent their respective transcriptomes (Fig. S5). The 6,615 differentially expressed genes were clustered into eight expression modules using WGCNA (Fig. 7 and Fig. S6).

10.1128/mSystems.00596-19.5 FIG S5 Rarefaction curve of the A. aegypti samples. A rarefaction curve was generated by taking a subset of counts for each of the *A. aegypti* samples and determining the number of protein-encoding genes able to be detected at each subset step. Each rarefaction curve is labeled by a color corresponding to a life stage. The circular points at the end of each curve represents the total number of protein-encoding genes detected with the full counts of the sample. Download FIG S5, TIF file, 0.8 MB. Copyright © 2019 Chung et al. 2019 Chung et al. This content is distributed under the terms of the Creative Commons Attribution 4.0 International license.

10.1128/mSystems.00596-19.6 FIG S6 Heatmap of the log_2_(TPM) of the differentially expressed A. aegypti genes. The heatmap shows the log_2_(TPM) values of the 6,653 differentially expressed A. aegypti genes from 18-hpi, 4-dpi, and 8-dpi samples along with their post-blood-feeding control counterparts. The horizontal color bar and the key above the heatmap indicate the samples represented in each of the heatmap columns. The leftmost vertical color bar on the left of the heatmap represents the nine different WGCNA expression modules generated using the 10,398 differentially expressed B. malayi genes. Within each individual expression module, there are two expression patterns that are the opposite of each other The inner vertical colored bar indicates whether the genes cluster with the main (gray) or inverse (black) expression pattern of the module. Download FIG S6, TIF file, 3.5 MB. Copyright © 2019 Chung et al. 2019 Chung et al. This content is distributed under the terms of the Creative Commons Attribution 4.0 International license.

Some clusters likely represent differential gene expression relevant to blood feeding. For instance, the third largest WGCNA cluster contains 780 A. aegypti genes upregulated at 18 h in both the infected and control mosquitoes (Fig. 7). Overrepresented functional terms identified in this module include vitellinogens, a family of secreted proteins with an important role in yolk formation (35); insect allergen proteins, a protein family found to be widespread in insects with roles in nutrient uptake and detoxification (36); and lipid transport proteins. Previous studies have shown the upregulation of genes encoding vitellogenin and insect allergen proteins to be a typical postfeeding response in mosquitoes (37, 38).

**(i) Eighteen hours postfeeding.** By 18 hpi, microfilariae ingested from an infected mammal have invaded the mosquito thoracic muscles and have begun to shorten to a distinct morphological form, commonly referred to as the L1 or sausage stage. There are 633 A. aegypti genes upregulated at this time point in only the infected mosquitoes (Fig. 7). The most significantly overrepresented functional terms involve proteins with oxidoreductase activity, roles in signal recognition particle-dependent protein targeting to the membrane, ATP synthesis-coupled proton transport, and the tricarboxylic acid cycle. Additionally, we observe an overrepresentation in structural constituents of the ribosome, specifically those associated with mitochondrial, rather than cytosolic, translation. Confirming previous observations, this enrichment of proton transport and ATP synthase activity at 18 hpi is an indicator of metabolic disturbance in an early response to B. malayi infection (22).

**(ii) Four days postinfection.** The 4-dpi samples mark the L1-to-L2 molt of B. malayi in the thoracic muscle. The largest A. aegypti expression module generated contains 2,574 genes upregulated specifically in the blood-fed control samples (Fig. 7). Overrepresented proteins in this cluster include helicases, zinc fingers, and proteins with bromodomain folds. Additionally, the cluster is overrepresented in kinases, phosphatases, and other proteins with roles in intracellular signal transduction. These transcriptional changes and triggered signal pathways are absent in nematode-infected mosquitoes, indicating that the continued development of B. malayi in the mosquito is interfering with normal metabolic activity. The smallest recovered A. aegypti expression module contained 51 genes differentially expressed in mosquitoes 4 days postinfection (Fig. 7). However, we observed no significantly overrepresented functional terms.

**(iii) Eight days postinfection.** The second largest module of A. aegypti differentially expressed genes includes 1,365 genes specifically upregulated at 8 days in infected A. aegypti, coinciding with the L2-to-L3 molt of B. malayi in the thoracic muscle (Fig. 7). As with previous studies, we observed an increase in α-crystallin heat shock proteins, a family of chaperones induced to cope with stressful conditions (22, 39), likely from the continued growth of B. malayi. However, while a previous study observed small heat shock proteins to have a temporal expression pattern at 1, 6, and 8 dpi of B. malayi, we observed detectable upregulation of these heat shock proteins only at 8 dpi (22, 39). Additionally, proteasomal components, including serine proteases, were identified to be overrepresented in this cluster of genes.

**(iv) Post-blood feeding versus postinfection.** In the fifth largest A. aegypti expression module, 453 genes were upregulated in only the noninfected, blood-fed control mosquitoes, while 190 genes were upregulated only in infected mosquitoes (Fig. 7). While no functional terms were overrepresented in upregulated genes from infected mosquitoes, the controls were overrepresented in translational proteins and structural constituents of the cytosolic ribosome as opposed to the structural constituents of the mitochondrial ribosome that were upregulated in the 18-hpi mosquitoes. We also recovered a module containing 89 genes upregulated in only the uninfected 4- and 8-day-post-blood-feeding (dpbf) mosquitoes that were significantly overrepresented in proteins with translation initiation factor activity (Fig. 7).

### B. malayi drug target identification and *in vitro* validation.

**FIG 8:**
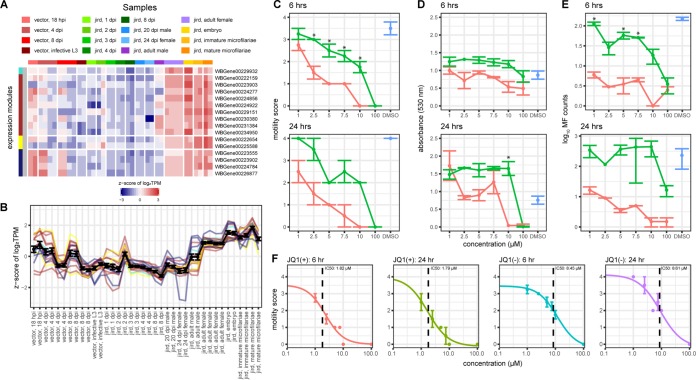
Targeting BET proteins in adult female B. malayi. (a) The heatmap shows the z-score of the log_2_(TPM) values of the 16 genes encoding bromodomain-containing proteins in the B. malayi genome. The horizontal color bar and the key above the heatmap indicate the samples represented in each of the heatmap columns. The vertical color bar on the left of the heatmap shows the assigned WGCNA expression clusters for each of the 16 genes. (B) The line plot shows the log_2_(TPM) values for each of the 16 bromodomain-containing genes across the B. malayi life cycle. Each individual line is colored according to the assigned WGCNA module of the specific gene as seen in panel A, while the black line represents the average log_2_(TPM) value of all 16 bromodomain-containing genes. Error bars represent the standard error of the mean. (C to E) The efficacy of JQ1(+) was assessed on adult female B. malayi at 6 h and 24 h using a motility scoring assay (C), the colorimetric MTT assay for viability measured at 530 nm (D), and microfilaria (MF) counts (E). Each point is indicative of two replicates, and the error bars indicate standard error of the mean. For each of the assays, the effect of 1, 2.5, 5, 7.5, 10, and 100 μM JQ1(+) (red) and its negative enantiomer JQ1(−) (green) was assessed along with DMSO (a vehicle control; blue). Asterisks indicate significant differences (*P* < 0.05, Student’s unpaired *t* test) between the JQ1(+) and JQ1(−) treatments. (F) IC_50_ curves were generated for 6 and 24 h for JQ1(+) and JQ1(−), with error bars representing the standard error of the mean.

Because current mass drug administration (MDA) programs for lymphatic filariasis are primarily microfilaricidal and have a limited efficacy on adult worms (6, 8), we sought to identify a potential adulticidal drug target using our transcriptomics data set. Upregulation of chromatin remodeling was frequently observed in modules associated with the adult female samples. Bromodomain-containing proteins interact with chromatin remodeling complexes to regulate transcription or to facilitate DNA repair protein accessibility (40), leading to their study in cancer biology as drug targets. Despite being significantly enriched in only one B. malayi expression cluster (Fig. 3), we find that all 16 bromodomain-containing proteins in the B. malayi genome are spread across four different modules that are all upregulated in the adult female, embryo, and microfilaria life stages (Fig. 8A and B), suggesting an underlying need for large-scale chromatin remodeling during these life stages.

JQ1(+) is a bromodomain inhibitor that binds to members of the bromodomain and extraterminal (BET) family of transcription factors (41). By competitively binding to BET proteins, JQ1(+) prevents BET proteins from binding to acetylated lysine residues on chromatin and recruiting transcription factors (42). JQ1(+) has been identified to be a promising cancer therapeutic, having been shown to prevent BRD4, a BET family protein, from interacting with and recruiting Myc, a transcription factor involved in cell proliferation that has been found to be constitutively active in several cancers, such as acute myeloid leukemia and multiple myeloma (42, 43).

Of the 16 bromodomain proteins encoded by the B. malayi genome, only two are members of the BET family of proteins and orthologs of the human and mouse BET proteins. Similarly, Caenorhabditis elegans has three reported BET orthologs: bet-1, bet-2, and F13C5.2. Although there were no observed RNA interference (RNAi) phenotypes for bet-2, knockdowns of both bet-1 and F13C5.2 were found to result in embryonic lethality, larval arrest, locomotion alterations, and sterility (44), indicating the importance of the C. elegans BET proteins in development and reproduction. Additionally, interfering with the activity of BET proteins has the potential to cause adult worm lethality, with knockdowns of bet-1 causing adult C. elegans lethality and knockdowns of F13C5.2 causing ruptures in the vulva of female C. elegans (44). Due to the potential importance of the BET proteins in B. malayi reproduction and sterility, we tested the efficacy of JQ1(+) on B. malayi.

Adult female B. malayi worms were treated with JQ1(+) to assess the effect of BET inhibition on worm viability. JQ1(+) concentrations ranging from 1 to 100 μM were used, similar to those used in *in vitro* human cancer studies (45, 46). The efficacy of the drug on the adult female worm was assessed at 6 and 24 h posttreatment using motility scoring and the colorimetric 3-(4,5-dimethylthiazolyl-2)-2,5-diphenyltetrazolium bromide (MTT) viability assay (48). At both time points, 10 and 100 μM JQ1(+) were found to cause complete loss of motility in adult female worms, while the negative enantiomer JQ1(−) caused complete loss of motility only at 100 μM (Fig. 8C). At 6 h, adult worms had decreased viability according to the MTT assay at 10 μM and 100 μM JQ1(+) relative to JQ1(−) and the dimethyl sulfoxide (DMSO) vehicle control (Fig. 8D). At 24 h, 10 and 100 μM JQ1(+) resulted in significantly reduced viability relative to the DMSO vehicle control. As such, JQ1(+) has adulticidal effects on filarial nematodes *in vitro*. Additionally, we assessed worm fecundity by measuring microfilariae shed from treated adult female worms. At 6 and 24 h, all concentrations of JQ1(+) resulted in reduced fecundity *in vitro* relative to JQ1(−) and the vehicle control DMSO, providing evidence that even 1 μM JQ1(+), the lowest tested concentration, may potentially sterilize adult female worms (Fig. 8E). At both 6 and 24 h, we observed that the 50% inhibitory concentration (IC_50_) of JQ1(+) is ∼1.8 μM (Fig. 8F), similar to the IC_50_s of other promising antifilaricidal therapeutics (49).

## DISCUSSION

### B. malayi transcriptome and drug target identification and *in vitro* validation.

This analysis of the B. malayi transcriptome reveals overarching transcriptional themes during critical life stages. The overrepresentation of kinases and phosphatases in adult male B. malayi indicates the presence of male-specific kinase signaling cascades. Conversely, adult female and embryo B. malayi stages are overrepresented in DNA-binding proteins with roles in chromatin remodeling, such as zinc fingers and helicases. This indicates that during the adult female, embryo, and microfilaria stages, significant chromatin remodeling is potentially required for reproduction and further development of B. malayi.

Proteins associated with chromatin remodeling, including those that contain bromodomains, may be a prospective new drug target for the development of adulticidal therapeutics. Our results with the BET inhibitor JQ1(+) indicate that it is more lethal than its chiral enantiomer JQ1(−), suggesting a specific effect and not just general toxicity. JQ1(+) treatment also reduced microfilaria output, suggesting that JQ1(+) could be microfilaricidal and/or sterilizing the adult female worms. Given that our studies are *in vitro*, our results with JQ1(+) are promising, but preliminary. While *in vitro* effects of an anthelminthic may not always correlate with *in vivo* activity, *in vitro* experiments are consistently used as a cost-effective method to identify promising drug candidates (49, 50). Future work needs to focus on (a) studies with lower concentrations which will likely refine and lower the IC_50_, (b) assessing microfilaricidal effects, and (c) assessing the efficacy of JQ1(+) in gerbils, measuring worm burden, development, and fecundity.

Although JQ1(+) was initially touted as a prospective cancer therapeutic, its low half-life in sera limits its efficacy as a cancer therapeutic (51); the required properties for lymphatic filariasis are unknown but likely to be different. Following oral administration of JQ1(+) at 10 mg/kg of body weight in CD1 mice, pharmacokinetic studies have measured: (a) bioavailability (*F* = 49%), (b) peak plasma concentration (*C*_max_ = 1,180 ng/ml), and (c) drug exposure (area under the concentration-time curve [AUC] = 2,090 h · ng/ml) with a time to maximum concentration of drug in serum (*T*_max_) of 0.25 h and half-life (*t*_1/2_) of 1.39 h (52). JQ1(+) has also been examined as a potential male contraceptive having higher testicular bioavailability (AUC_testes_/AUC_plasma_ = 259%), with pronounced (*C*_max_ = 34 μg/ml) and rapid (*T*_max_ = 0.25 h) exposure (53). Higher testicular bioavailability could be potentially beneficial in treating hydrocele formed by lymphatic filariasis. A number of BET inhibitors with alternative pharmacokinetic properties are currently in clinical trials as cancer therapeutics (42); future studies could test these derivatives for the treatment of lymphatic filariasis.

### *w*Bm transcriptome.

In contrast to published multispecies transcriptomes of filarial *Wolbachia* endosymbionts across their invertebrate host’s life cycles (19, 54), the *w*Bm transcriptome across the life cycle had statistically robust gene expression but with very weak effects. Compared to other studies analyzing the transcriptome of *w*Bm (19) and *w*Di (54), the latter of which is the *Wolbachia* endosymbiont of Dirofilaria immitis, we obtained a greater number of reads mapping to protein-encoding regions compared to the 2017 *w*Bm transcriptome study (6,178 to 31,289 reads mapping to protein-encoding genes) (19); the 2014 *w*Di transcriptome study (495 to 27,118 reads) (54); this study with the poly(A), rRNA depletion (∼8,525 to 284,943 reads); and this study with the Agilent SureSelect enrichment (240,693 to 35,276,890 reads).

The vector samples were prepared using the Agilent SureSelect enrichment instead of the standard poly(A) and rRNA depletion such that genes could potentially cluster based on the different enrichment protocols rather than biological differences. However, we previously demonstrated that samples prepared using the Agilent SureSelect enrichment did not impart a significant bias relative to their poly(A)- and rRNA-depleted counterpart (24). Furthermore, we do not see similar profiles for all our samples prepared using the Agilent SureSelect enrichment (Fig. 5A).

Additionally, there are differences in the annotation used between studies on the *w*Bm transcriptome. Our study uses the RefSeq annotation, which has two distinct annotations for the 6S RNA and the adjacent gene *Wbm0439*, while the original published annotation (55) does not report the 6S RNA and instead uses an upstream start codon for *Wbm0439* that extends into the 6S RNA. As such, the 2017 *w*Bm transcriptome study (19), which used the original published annotation, reported the differential expression of *Wbm0439* as adult female worms mature, but we now understand from using the RefSeq annotation that this observed increase in expression likely originates from the 6S RNA and not *Wbm0439*.

Although the absence of the heme, riboflavin, and flavin adenine dinucleotide (FAD) biosynthetic pathways in the B. malayi genome has generated hypotheses that *w*Bm provides these needed metabolites to B. malayi by upregulating endosymbiont gene expression (16), both this and the 2017 *w*Bm transcriptome study suggest that these pathways may be constitutively expressed in *w*Bm. While there may be specific *w*Bm genes that are differentially expressed, we see little indication of strong transcriptional responses from global regulation of gene expression. Despite strong statistical support for differential expression, the transcriptional response (or effect) is weak.

**FIG 9:**
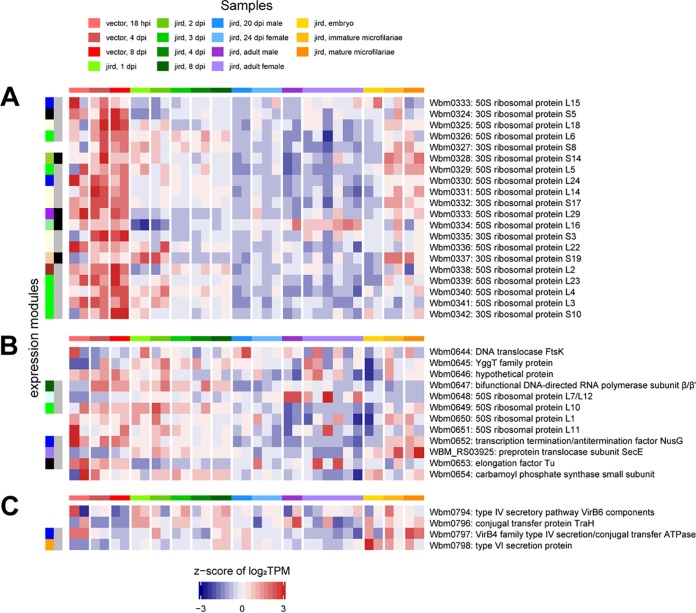
*w*Bm WGNCA expression modules of three putative operons. The heatmap shows the z-score of the log_2_(TPM) values of three putative *w*Bm operons encoding 20 ribosomal proteins (A), 4 ribosomal proteins and other housekeeping genes (B), and the type IV secretion system proteins (C). The horizontal color bar above the heatmap indicates the samples represented in each of the heatmap columns according to the key at the top. The vertical color bars on the left of the heatmap illustrate the WGCNA expression modules in Fig. 5 generated using the differentially expressed *w*Bm genes. Genes with no vertical bar indicate that the gene is not differentially expressed.

As a bacterial endosymbiont, *w*Bm is expected to have operons that should confound analyses like WGCNA since the measures of transcription are not likely to be independent for adjacent genes. Yet, for three putative operons of likely cotranscribed genes, which should be in the same module, the genes are placed in disparate modules or lack statistically significant differential expression (Fig. 9). We propose that rather than altering the expression of specific metabolic pathways across the B. malayi life cycle, *w*Bm may primarily alter its rates of transcription and translation via the 6S RNA. The original genome analyses of *w*Bm and *w*Mel (the *Wolbachia* endosymbiont of Drosophila melanogaster) both noted few transcriptional regulators and suggested that genes may be constitutively expressed (55, 56) such that minimal global regulation may not be surprising and may reflect the limited environmental variation for obligate intracellular bacteria. Despite the lack of a strong effect from global regulation of gene expression in *w*Bm across the life cycle, it is possible that there is global regulation of gene expression within different populations of B. malayi cells (e.g., oocytes or spermatocytes) or a weak transcriptional response that can be better observed with many more independent biological replicates than were feasible in this study.

### A. aegypti transcriptome.

In A. aegypti, our data show that parasitism by B. malayi has severe effects on transcription related to normal homeostatic processes. At 18 hpi, genes encoding proteins with roles in ATP-coupled protein transport and mitochondrial translation were elevated, likely due to increased energy demands by infected mosquitoes. This upregulation coincides with previous observations of the breakdown of mitochondria and the depletion of microfiber glycogen reserves upon nematode infection of A. aegypti (57). Additionally, the absence of upregulated vitellogenin and insect allergen-related proteins in the 18-hpi samples suggests a decrease in A. aegypti fecundity upon the uptake of B. malayi (58, 59). At 4 dpi, coinciding with the L1-to-L2 molt of B. malayi, there is a remarkable upregulation of many genes in the control mosquitoes that were not present in the infected mosquitoes. These include genes encoding proteins with roles in transcriptional regulation, such as zinc fingers and bromodomains, along with proteins with roles in signaling cascades, such as kinases, phosphatases, and intracellular signal transduction proteins. These large perturbations in the A. aegypti transcriptome suggest a large impact on vector host viability by B. malayi.

### Conclusions.

Overall, this study has led to the discovery of BET inhibitors as potential new therapeutics for lymphatic filariasis worthy of further investigation and development. Additionally, our multispecies RNA-Seq analysis across the B. malayi life cycle serves as a comprehensive data set that can enable the identification of other therapeutics and address other questions regarding the interplay between the different organisms in the B. malayi life cycle.

## MATERIALS AND METHODS

### Sample preparation—nematodes.

Third-stage larvae of Brugia malayi strain FR3 were isolated from infected mosquitoes 9 to 16 days postinfection (dpi) using the NIAID/NIH Filariasis Research Reagent Resource Center (FR3) Research Protocol 8.4 (http://www.filariasiscenter.org/protocols/Protocols; 60). Larvae were isolated in RPMI plus P/S, enumerated, and either flash frozen in liquid nitrogen for storage at −80°C or used for the infection of male Mongolian gerbils (Meriones unguiculatus; Charles River, Wilmington, MA, USA). Gerbils were anesthetized with 5% isoflurane and infected by introducing 400 L3s into the peritoneal cavity using a butterfly catheter. Larvae, immature adults, and mature adults were recovered by peritoneal flush using a terminal procedure. Brugia malayi embryos were collected from live gravid females as previously described (20).

Generation of immature and mature microfilariae required the surgical transplantation of adult B. malayi into the peritoneal cavity of recipient gerbils. Microfilariae were isolated either through a peritoneal tap (61) or by terminal worm recovery. For terminal worm recoveries, gerbils were euthanized by CO_2_ asphyxiation followed by cervical dislocation. The peritoneal cavities were then opened and soaked with RPMI plus P/S. All animal care and use protocols were approved by the University of Wisconsin Oshkosh IACUC. Nematode preparations were flash frozen in liquid nitrogen and stored at −80°C prior to RNA isolation.

### Sample preparation—mosquitoes.

The Aedes aegypti black-eyed Liverpool strain was obtained from FR3 and maintained in the Biosafety Level 2 Insectary at the University of Wisconsin Oshkosh. Desiccated mosquito eggs were hatched in deoxygenated water, and the resulting larvae were maintained on a slurry of ground TetraMin fish food (Blacksburg, VA) at 27°C with 80% relative humidity. Female pupae were separated using a commercial larva pupa separator (The John Hock Company, Gainesville, FL) and placed in mesh-covered paper soup cartons, and eclosed adults were fed with cotton pads soaked in sucrose solution. Females were deprived of sucrose approximately 12 h prior to blood feeding. Mosquitoes were infected with the Brugia malayi FR3 strain by feeding microfilaremic cat blood (FR3) through Parafilm via a glass-jacketed artificial membrane feeder. When necessary, microfilaremic cat blood was diluted with uninfected dog blood to achieve a suitable parasite density for infection (100 to 250 microfilariae/20 μl). Infected mosquitoes were collected at 18 h postinfection (hpi), 4 dpi, and 8 dpi. They were anesthetized with CO_2_ and then transferred to chilled microscope slides, where the legs, wings, heads, and abdomens were removed. The thoraces with and without developing B. malayi larvae were flash frozen in liquid nitrogen and stored at −80°C prior to RNA isolation. Vector-derived infective B. malayi L3s were isolated from whole mosquitoes in bulk using the standard FR3 protocol (60).

### RNA isolation.

Mosquito thoraces were combined with TRIzol (Zymo Research, Irvine, CA) at a ratio of 1 ml TRIzol per 50 to 100 mg mosquito tissue, while nematode samples were processed using a 3:1 volume ratio of TRIzol to sample. For both preparations, 1 μl β-mercaptoethanol was added for every 100 μl of sample. The tissues were homogenized using a bead beater and a TissueLyser (Qiagen, Germantown, MD) at 50 Hz for 5 min. The homogenate was then transferred to a new tube and centrifuged at 12,000 × *g* for 10 min at 4°C. After incubation at room temperature for 5 min, 0.2 ml chloroform was added for every 1 ml TRIzol. The samples were shaken by hand for 15 s, incubated at room temperature for 3 min, loaded into a prespun Phase Lock Gel heavy tube (5Prime, Gaithersburg, MD), and centrifuged at 12,000 × *g* for 5 min at 4°C. The upper phase was extracted, and 1 volume of 100% ethanol was added and then loaded onto a PureLink RNA Mini column (Ambion, Austin, TX). The samples were processed according to the manufacturer’s instructions, quantified using a Qubit fluorometer (Qiagen, Germantown, MD) and NanoDrop spectrometer (NanoDrop, Wilmington, DE), and sent to the Institute for Genomic Sciences at the University of Maryland Baltimore for DNase treatment and library preparation.

### Library preparation and sequencing.

Transcriptome sequencing was conducted as previously described (62). Briefly, whole-transcriptome libraries without AgSS capture were constructed for sequencing on the Illumina platform using the NEBNext Ultra Directional RNA Library Prep kit (New England Biolabs [NEB], Ipswich, MA, USA). For targeting eukaryotic mRNA, polyadenylated RNA was isolated using the NEBNext poly(A) mRNA magnetic isolation module. When targeting bacterial mRNA, samples underwent rRNA and poly(A) reductions, as previously described (23, 63). SPRIselect reagent (Beckman Coulter Genomics, Danvers, MA, USA) was used for cDNA purification between enzymatic reactions and size selection. The PCR amplification step was performed with primers containing a 7-nucleotide (nt) index sequence. Libraries were evaluated using the GX touch capillary electrophoresis system (Perkin-Elmer, Waltham, MA) and sequenced on an Illumina HiSeq2500 sequencer, generating 100-bp paired-end reads.

For the B. malayi and *w*Bm AgSS capture designs, probes were designed using the SureSelect DNA Advanced Design Wizard for B. malayi and *w*Bm (24). Probes were designed to capture every 120 bp for each coding sequence in both organisms with no overlap between baits. DustMasker was used to identify and mask low-complexity regions of the genome from the probe design.

For samples treated with the *w*Bm AgSS capture, precapture libraries were constructed from 500 to 1,000 ng of total RNA samples using the NEBNext Ultra Directional RNA Library Prep kit (NEB, Ipswich, MA, USA). First-strand cDNA was synthesized without mRNA extraction to retain nonpolyadenylated transcripts and fragmented at 94°C for 8 min. After adaptor ligation, cDNA fragments were amplified with 10 cycles of PCR before capture. *Wolbachia* transcripts were captured from 200 ng of the amplified libraries using the Agilent SureSelectXT RNA (0.5- to 2-Mbp) bait library designed specifically for *w*Bm. Library-bait hybridization reaction mixtures were incubated at 65°C for 24 h and then bound to MyOne streptavidin T1 Dynabeads (Invitrogen, Carlsbad, CA, USA). After multiple washes, bead-bound captured library fragments were amplified with 18 cycles of PCR. The libraries were loaded on an Illumina HiSeq4000, generating 151-bp paired-end reads.

For samples treated with the B. malayi AgSS capture, precapture libraries were constructed from 1000 ng of total RNA samples using the NEBNext Ultra Directional RNA Library Prep kit (NEB, Ipswich, MA, USA). After adaptor ligation, cDNA fragments were amplified for 10 cycles of PCR before capture. B. malayi transcripts were captured from 200 ng of the amplified libraries using an Agilent SureSelectXT custom (12- to 24-Mbp) bait library designed specifically for B. malayi. Library-bait hybridization reaction mixtures were incubated at 65°C for 24 h and then bound to MyOne streptavidin T1 Dynabeads (Invitrogen, Carlsbad, CA). After multiple rounds of washes, bead-bound captured library fragments were amplified with 16 cycles of PCR. The libraries were loaded on an Illumina HiSeq 4000, generating 151-bp paired-end reads.

### RNA-Seq analyses.

For all samples used in the analysis, sequencing reads were mapped to the B. malayi genome WS259 (www.wormbase.org) using the splice junction mapper TopHat v1.4.0 (64) and the *w*Bm genome (55) using the Bowtie v0.12.9 aligner (65). All vector samples were aligned to the A. aegypti Liverpool strain genome AaegL3.3 (www.vectorbase.org) using TopHat v1.4.0 (64). Feature counts for the B. malayi and A. aegypti alignments were calculated using the union mode of HTSeq v0.5.3p9 (66) for all exon features. Feature counts for *w*Bm were calculated using the prokaryotic feature counter FADU (67) using the annotation provided by NCBI (NC_006833.1) (see Table S1 in the supplemental material). Using RNAmmer v1.2 (68), we identified five protein-encoding genes in the B. malayi WS259 annotation that overlapped with predicted rRNAs: WBGene00228061, WBGene00268654, WBGene00268655, WBGene00268656, and WBGene00268657. These five genes were excluded from differential expression analyses. Rarefaction analyses were done using the R package vegan using the counts for each organism. All samples were used for the transcriptomics analysis with the exception of the poly(A)-selected vector 18-hpi samples for B. malayi due to the rarefaction analysis indicating an insufficient number of reads.

10.1128/mSystems.00596-19.7 TABLE S1 Enrichment techniques and mapping statistics for individual samples. Download Table S1, XLSX file, 0.02 MB. Copyright © 2019 Chung et al. 2019 Chung et al. This content is distributed under the terms of the Creative Commons Attribution 4.0 International license.

Differentially expressed genes across all samples were identified for each organism individually by processing the read counts for all protein-encoding genes using edgeR v3.20.1 (69). Within edgeR, read counts were normalized and filtered so that only genes with a count-per-million (cpm) value equivalent to 5 reads per gene in the lowest sequenced sample in at least two samples would be kept. The qCML common dispersion and tagwise dispersions were estimated for the counts of each organism and fitted to a generalized linear model. Differentially expressed genes were determined using a quasilikelihood *F* test and defined as genes with a *P* value and false-discovery rate (FDR) of <0.05.

### WGCNA module detection and functional term enrichments.

Read counts were converted to transcript-per-million (TPM) values to normalize the gene expression across samples. For each organism individually, the TPM values for differentially expressed genes were processed using the R package WGCNA v1.61 (25) to identify expression modules across the B. malayi life cycle. For the soft thresholds for each expression subset, values of 5, 6, and 7 were chosen for B. malayi, *w*Bm, and A. aegypti, respectively. Modules were derived from each organism’s data set by hierarchically clustering genes based on dissimilarity in a topological overlap matrix and using a dynamic tree cut at a height that encompasses 99% of the truncated height range in the observed dendrogram (minimum cluster size, 1). Closely related modules were merged using a merge eigengene dissimilarity threshold of 0.25, 0.25, and 0.40 for B. malayi, *w*Bm, and A. aegypti, respectively. Each individual expression module was further divided into two clusters depending on whether the expression pattern of a gene has a higher Pearson correlation to the eigengene or the inverse eigengene.

InterPro descriptions and gene ontology (GO) terms for each of the protein-encoding genes in the A. aegypti, B. malayi, and *w*Bm genomes were obtained using InterProScan v5.22.61.0 (70). Overrepresented terms for each WGCNA module were defined as terms with a Fisher exact test FDR of <0.05.

### B. malayi response to JQ1(+).

Adult female B. malayi worms for testing JQ1(+) were supplied by TRS Laboratories (Athens, GA, USA) in RPMI culture medium. The experiment was conducted under a laminar flow hood sprayed with 70% ethanol before use. Upon arrival, worms were placed in prewarmed 37°C sterile RPMI 1640 medium (HyClone, Logan, UT, USA) supplemented with final concentrations of 5 g/liter glucose, 10% fetal bovine serum, 2 mM l-glutamine, 100 U/ml penicillin, 100 μg/ml streptomycin, and 250 ng/ml amphotericin (Sigma-Aldrich, MO, USA) and incubated for 3 to 4 h at 37°C in 5% CO_2_.

After acclimation, worms were exposed to different drug concentrations obtained from stock solutions of 100 mM JQ1 dissolved in 100% DMSO. Worms were transferred to 12-well plates (2 worms per well, 2 well replicates per treatment) with each well containing 990 μl of the glucose-, serum-, and antibiotic-supplemented RPMI medium and 10 μl of JQ1(+), JQ1(−), and/or DMSO to obtain the final concentration tested, such that all wells had a final concentration of 1% DMSO. Worms were kept at 37°C in 5% CO_2_, and their viability was assessed at 6 and 24 h for each treatment using worm motility, microfilaria release, and the MTT reduction assay at an absorbance of 530 nm (48). The MTT reduction assay relies on the ability of living organisms to reduce tetrazolium MTT [3-(4,5-dimethylthiazolyl-2)-2,5-diphenyltetrazolium bromide] to formazan. Formazan is then dissolved in DMSO, and its absorbance at 530 nm is assessed. Adult female B. malayi worms were treated with 1, 2.5, 5, 7.5, 10, and 100 μM JQ1(+) drug and JQ1(−), its chiral enantiomer, *in vitro* to assess the effect of BET inhibition on their viability. DMSO functioned as a vehicle control for all drug experiments. Motility scores, microfilaria counts, and absorbances between JQ1(+)-treated worms were compared to DMSO using an unpaired Student's *t* test.

### Data availability.

The data set(s) supporting the results of this article is available in the Sequence Read Archive (SRA) repository. The A. aegypti and B. malayi sequencing reads are available in SRP068692, and the *w*Bm sequencing reads are available in SRP068711. Data files are available in GEO under the accession number GSE139965. Supplemental data files and source code for reproducing the transcriptomics analyses and figures can be downloaded from https://github.com/Dunning-Hotopp-Lab/lf_transcriptome. All code is made available under the MIT License.
