# Supplementary figures and images for: Drug Repurposing of Bromodomain Inhibitors as Potential Novel Therapeutic Leads for Lymphatic Filariasis Guided by Multispecies Transcriptomics
Source: mSystems. 2019 Dec 3;4(6):e00596-19. doi: 10.1128/mSystems.00596-19 (PMC6890932; doi:10.1128/mSystems.00596-19)

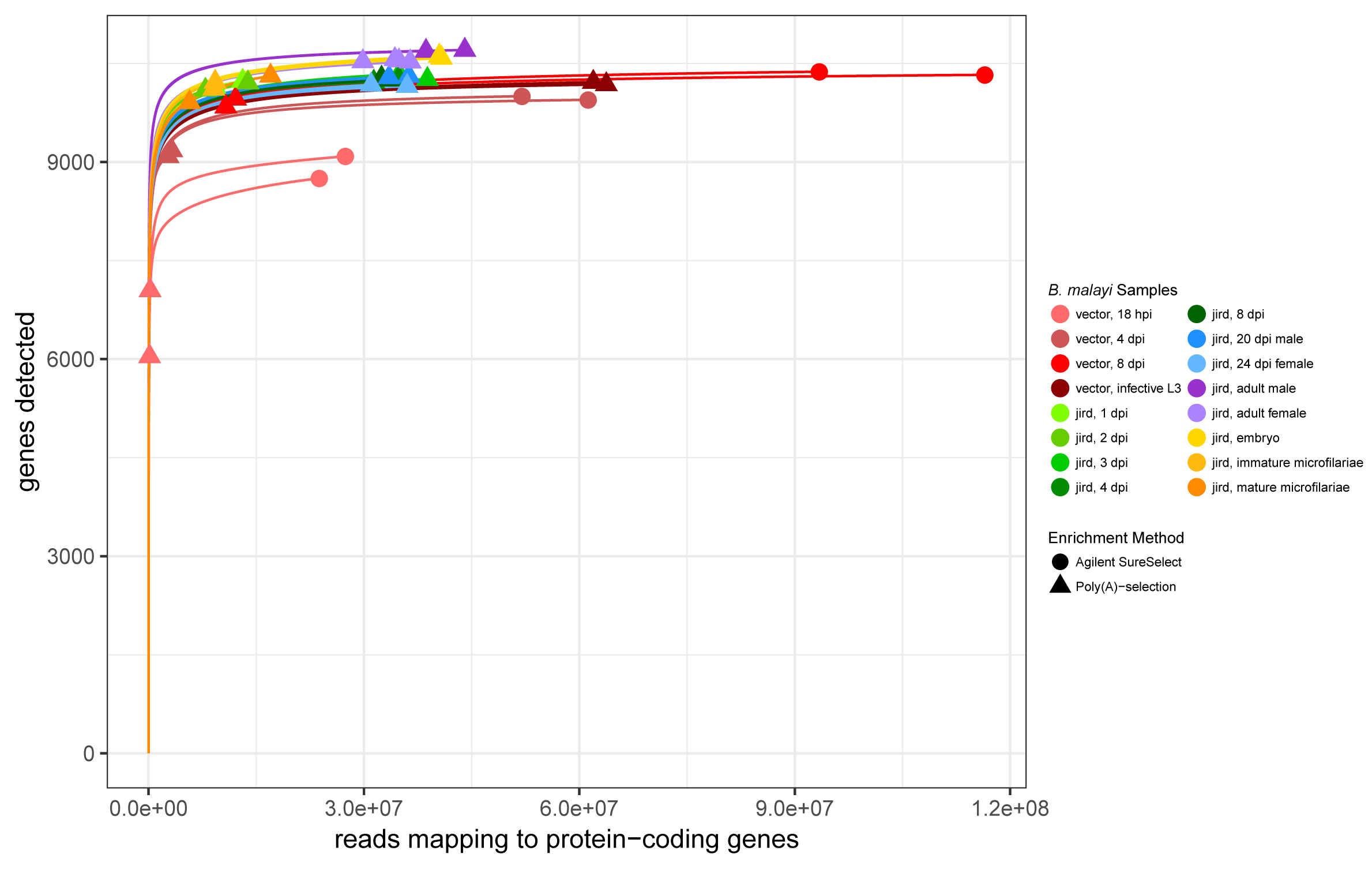

Supplement: FIG S1 [file mSystems.00596-19-sf001.tif]

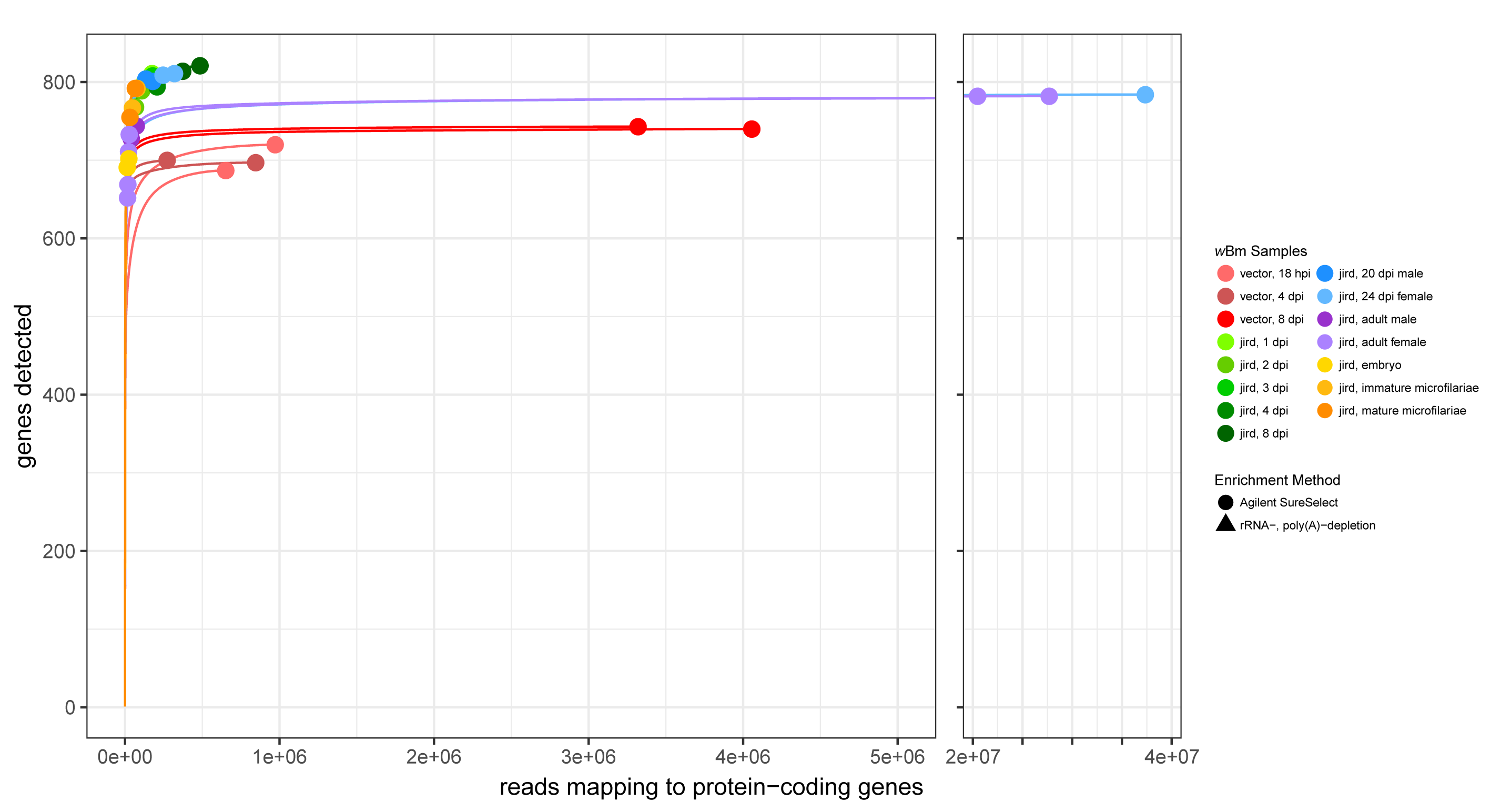

Supplement: FIG S3 [file mSystems.00596-19-sf003.tif]

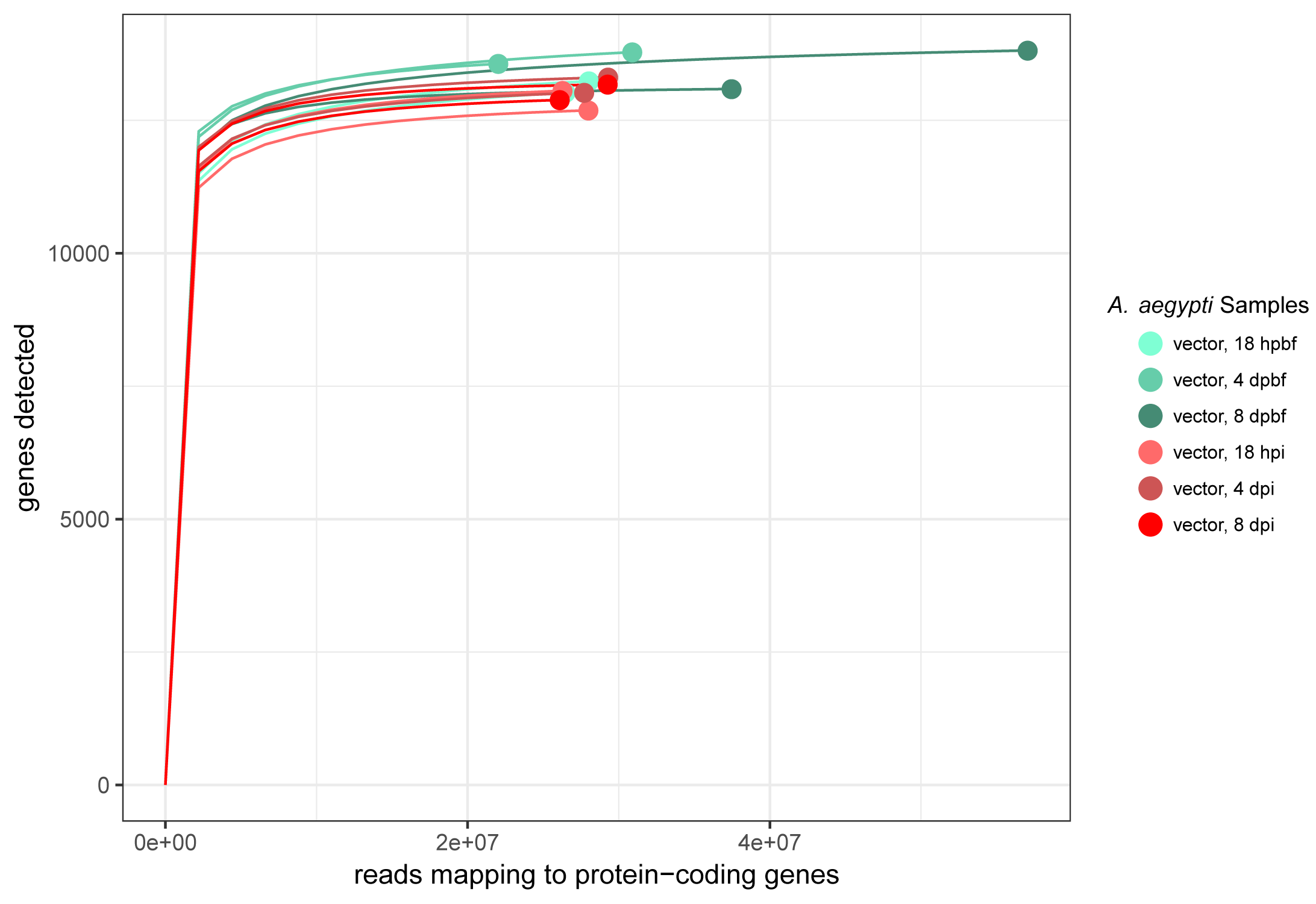

Supplement: FIG S5 [file mSystems.00596-19-sf005.tif]

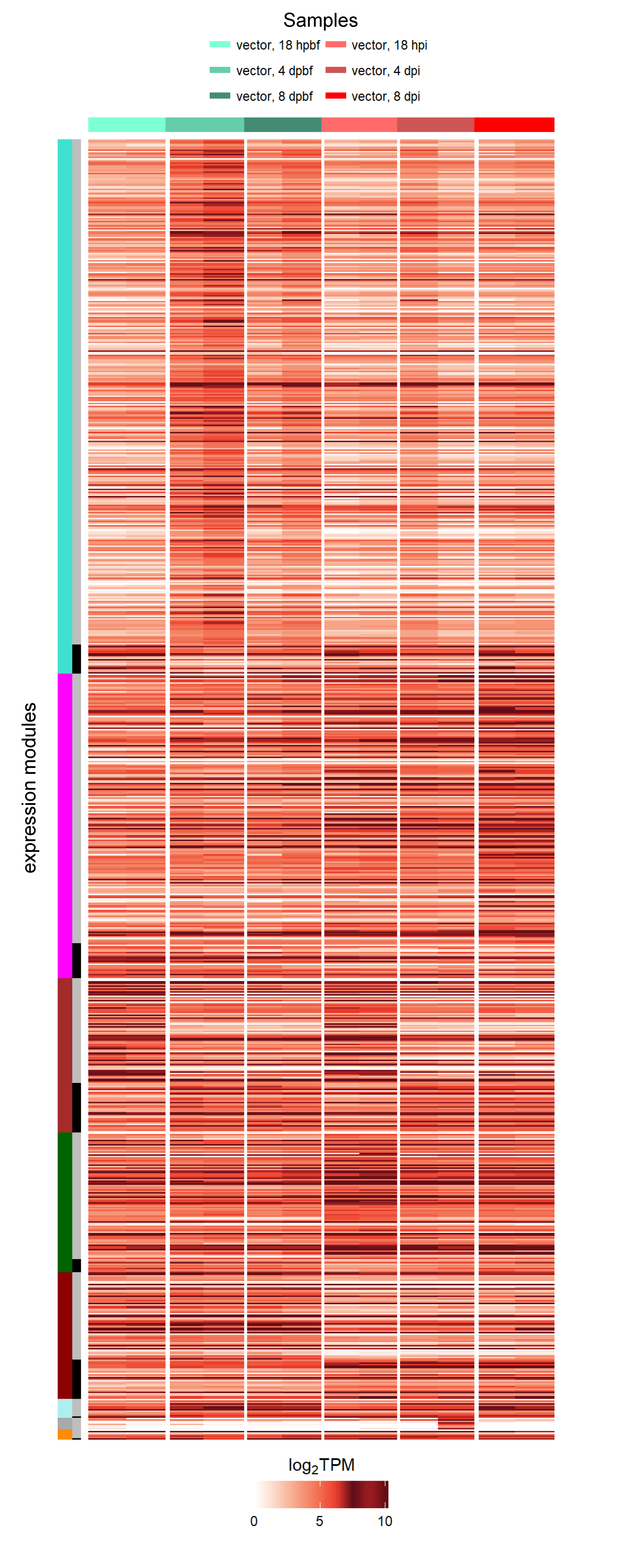

Supplement: FIG S6 [file mSystems.00596-19-sf006.tif]
